# Supplementary material for: A Likelihood Approach for Real-Time Calibration of Stochastic Compartmental Epidemic Models
Source: PLoS Comput Biol. 2017 Jan 17;13(1):e1005257. doi: 10.1371/journal.pcbi.1005257 (PMC5240920; doi:10.1371/journal.pcbi.1005257)
Supplement: S1 File — (TAR.GZ) [file pcbi.1005257.s014.tar.gz › HSPH_Online-SI-Revision/IPoi09/n10-mild/n10-mild_BENCH_table-4.pdf]

1	R0	{1.25046, 6.80278}	{1.55565, 9.30239}	{{1.54207, 9.08517}, {1.54207, 9.08517}}	{24.4066, 36.744}	inf	157.	511.333	{79., 748.}	245.648	0.542857	newly	177.	401.933	{49., 583.}	150.207	0.435786	Reff	1.16118	1.22401	{0.904887, 1.5029}	16.2563	ar	3516.	6240.27	{5907., 6434.}	77.482
2	R0	{1.32013, 4.45847}	{1.69254, 7.56319}	{{1.47651, 5.4396}, {1.87476, 9.28761}}	{28.2105, 69.6366}	inf	184.	541.907	{230., 957.}	196.638	0.417996	newly	307.	568.209	{288., 916.}	91.6749	0.25871	Reff	1.21267	1.41828	{1.12111, 1.70097}	21.0866	ar	4420.	6866.86	{5921., 7724.}	55.3588
3	R0	{1.31785, 5.36796}	{1.27121, 1.91565}	{{1.27121, 1.91565}, {1.27121, 1.91565}}	{3.5391, 64.3133}	inf	142.	136.619	{50., 256.}	45.9759	0.237348	newly	232.	392.	{147., 708.}	91.7898	0.282046	Reff	1.23206	0.862528	{0.787896, 0.92417}	29.9929	ar	4942.	3857.24	{3485., 4233.}	21.9499
4	R0	{1.38801, 6.16031}	{1.45244, 5.63669}	{{1.42659, 5.32185}, {1.454, 5.65591}}	{4.64178, 10.0961}	inf	202.	462.385	{172., 620.}	134.108	0.356302	newly	273.	588.103	{260., 798.}	118.24	0.32369	Reff	1.26267	1.0929	{0.931142, 1.32241}	15.0525	ar	4790.	5430.67	{5134., 5657.}	13.3751
5	R0	{1.27955, 11.57}	{1.51437, 16.2995}	{{1.51752, 16.3537}, {1.51752, 16.3537}}	{18.3517, 40.8771}	inf	185.	527.667	{365., 674.}	188.629	0.451707	newly	121.	225.963	{149., 291.}	90.7867	0.275099	Reff	1.13842	0.974778	{0.781371, 1.30841}	20.4736	ar	3460.	5918.56	{5649., 6090.}	71.0565
6	R0	{1.22184, 4.34395}	{1.43836, 3.42536}	{{1.43579, 3.38828}, {1.43579, 3.38828}}	{17.7205, 21.1465}	inf	108.	424.308	{164., 593.}	307.835	0.720933	newly	215.	852.769	{487., 1141.}	311.413	0.74234	Reff	1.17138	1.09076	{0.905553, 1.33902}	13.5623	ar	3524.	5227.77	{5095., 5739.}	55.9591
7	R0	{1.23856, 4.59068}	{1.40116, 4.24222}	{{1.40116, 4.24222}, {1.40116, 4.24222}}	{13.1281, 7.5906}	inf	160.	403.423	{247., 533.}	152.572	0.386684	newly	265.	646.846	{436., 779.}	144.093	0.375951	Reff	1.13155	1.02621	{0.859892, 1.22223}	13.138	ar	3855.	5127.88	{4843., 5417.}	33.0191
8	R0	{1.34531, 10.6689}	{1.5671, 8.88318}	{{1.56097, 8.77207}, {1.56097, 8.77207}}	{16.486, 16.7376}	inf	312.	648.	{392., 858.}	112.692	0.322514	newly	198.	509.133	{330., 641.}	160.202	0.405919	Reff	1.20365	1.08127	{0.825753, 1.36694}	17.304	ar	4852.	6281.17	{5944., 6552.}	29.4552
9	R0	{1.31775, 5.19226}	{1.42263, 5.16608}	{{1.41571, 4.94504}, {1.42659, 5.32185}}	{7.95865, 3.68566}	inf	120.	418.387	{229., 559.}	248.817	0.513802	newly	202.	581.742	{352., 754.}	187.991	0.437609	Reff	1.22551	1.07337	{0.887509, 1.29453}	14.9353	ar	4708.	5277.16	{5014., 5443.}	12.0892
10	R0	{1.25916, 6.89207}	{1.38889, 4.25615}	{{1.38889, 4.25615}, {1.38889, 4.25615}}	{10.3029, 38.2457}	inf	175.	208.769	{79., 372.}	48.1758	0.198407	newly	191.	305.	{102., 519.}	77.1647	0.240567	Reff	1.10151	0.795172	{0.695695, 0.904862}	28.3116	ar	3823.	5078.08	{4758., 5365.}	32.8296
11	R0	{1.20377, 5.46566}	{1.31023, 3.04996}	{{1.31023, 3.04996}, {1.31023, 3.04996}}	{8.84388, 44.1978}	inf	147.	239.647	{171., 325.}	67.507	0.216193	newly	200.	532.706	{290., 747.}	166.353	0.403539	Reff	1.11577	0.988453	{0.834092, 1.13427}	12.7846	ar	3114.	4271.65	{3848., 4506.}	37.1756
12	R0	{1.27878, 2.55804}	{1.81304, 7.71903}	{{1.54465, 5.30296}, {2.41896, 12.8735}}	{41.7792, 201.756}	inf	57.	249.804	{54., 528.}	352.494	0.605243	newly	277.	308.765	{60., 667.}	64.9253	0.340762	Reff	1.24259	1.78001	{1.34807, 2.4042}	43.4413	ar	3930.	7283.	{6016., 8845.}	85.3181
13	R0	{1.41227, 5.45696}	{2.095, 17.5037}	{{1.97176, 15.6958}, {2.2194, 19.1478}}	{48.3425, 220.759}	inf	188.	782.083	{148., 1366.}	326.108	0.56862	newly	295.	347.833	{74., 593.}	61.6836	0.299268	Reff	1.28587	1.72893	{1.33833, 2.07389}	36.0075	ar	4822.	8213.67	{7874., 8559.}	70.3373
14	R0	{1.32917, 7.27593}	{1.81177, 11.5978}	{{1.71608, 9.75208}, {1.86902, 12.6353}}	{36.3087, 59.3994}	inf	250.	871.46	{392., 1168.}	250.648	0.511631	newly	248.	567.2	{285., 732.}	132.71	0.351504	Reff	1.18482	1.32764	{1.02215, 1.63577}	18.0115	ar	4758.	7333.88	{6996., 7562.}	54.1379
15	R0	{1.31629, 9.97481}	{1.34961, 4.77879}	{{1.31748, 4.25564}, {1.3618, 4.98002}}	{2.55716, 52.0914}	inf	186.	245.686	{132., 369.}	48.2488	0.207877	newly	149.	345.886	{170., 533.}	141.572	0.387411	Reff	1.22178	0.962649	{0.770779, 1.22552}	22.3532	ar	4543.	4616.26	{4199., 4972.}	5.41996
16	R0	{1.36735, 5.74166}	{1.4421, 5.50817}	{{1.42659, 5.32185}, {1.454, 5.65591}}	{5.4668, 8.38441}	inf	182.	460.074	{152., 773.}	159.015	0.392166	newly	266.	586.852	{241., 778.}	124.826	0.339714	Reff	1.26507	1.13107	{0.922174, 1.32987}	11.9149	ar	4516.	5465.15	{5052., 6244.}	21.0175
17	R0	{1.27524, 13.2396}	{1.66934, 14.9193}	{{1.44641, 10.5617}, {1.87294, 19.0269}}	{30.9043, 21.41}	inf	80.	160.174	{12., 367.}	131.087	0.403249	newly	45.	86.6522	{6., 185.}	125.024	0.395669	Reff	1.24591	1.60762	{1.41459, 1.80402}	29.0322	ar	3731.	6733.59	{5612., 7474.}	80.4767
18	R0	{1.21884, 7.71545}	{1.32082, 4.80952}	{{1.28596, 3.98513}, {1.34002, 5.27199}}	{8.36668, 37.6638}	inf	167.	216.773	{68., 324.}	55.5525	0.245872	newly	159.	296.864	{81., 430.}	107.061	0.347234	Reff	1.1234	0.91093	{0.753444, 1.14384}	20.5834	ar	3080.	4480.91	{4049., 4818.}	45.4841
19	R0	{1.34879, 6.71464}	{1.51574, 4.88172}	{{1.50982, 4.79544}, {1.54465, 5.30296}}	{12.3778, 27.2974}	inf	339.	529.567	{326., 684.}	61.0718	0.200797	newly	410.	687.1	{393., 947.}	71.2276	0.220492	Reff	1.15092	0.900189	{0.725016, 1.13327}	24.138	ar	4585.	5971.37	{5673., 6259.}	30.237
20	R0	{1.37284, 5.75036}	{1.54035, 5.27114}	{{1.54465, 5.30296}, {1.54465, 5.30296}}	{12.2014, 8.33378}	inf	216.	598.143	{333., 759.}	176.918	0.4272	newly	303.	812.429	{541., 983.}	168.128	0.419603	Reff	1.23597	1.09782	{0.844924, 1.37628}	15.2724	ar	4750.	6095.68	{5874., 6326.}	28.3301
21	R0	{1.29938, 5.36419}	{1.95511, 10.1338}	{{1.87476, 9.18466}, {1.96658, 10.2409}}	{50.4647, 88.9151}	inf	198.	770.417	{180., 1342.}	296.978	0.545937	newly	270.	612.483	{184., 983.}	139.944	0.370041	Reff	1.18387	1.58736	{1.1753, 1.88234}	34.8357	ar	4337.	7850.37	{7582., 8069.}	81.0091
22	R0	{1.31882, 7.47626}	{1.95129, 14.0883}	{{1.78225, 11.7348}, {2.20181, 17.7758}}	{47.9572, 88.4404}	inf	227.	716.443	{298., 1119.}	223.095	0.482344	newly	235.	402.934	{179., 629.}	86.4597	0.274944	Reff	1.193	1.62047	{1.28913, 1.89497}	36.3661	ar	3977.	7820.89	{7326., 8454.}	96.6529
23	R0	{1.26215, 3.46375}	{2.69289, 16.6523}	{{2.19696, 12.0512}, {2.97157, 19.3547}}	{113.358, 380.759}	inf	117.	774.479	{225., 1468.}	566.594	0.737186	newly	283.	420.648	{113., 764.}	78.1615	0.291736	Reff	1.18314	2.33863	{1.8504, 2.81437}	97.663	ar	3607.	9109.08	{8795., 9394.}	152.539
24	R0	{1.16712, 4.19464}	{1.77856, 9.76872}	{{1.71337, 8.80249}, {1.81647, 10.5184}}	{52.3892, 132.886}	inf	122.	856.92	{311., 1181.}	606.328	0.807324	newly	227.	630.98	{317., 819.}	186.529	0.459276	Reff	1.04609	1.16706	{0.810767, 1.5951}	26.0205	ar	3000.	7196.3	{6927., 7436.}	139.877
25	R0	{1.22751, 3.34517}	{2.58208, 14.6013}	{{2.00349, 9.42599}, {2.96978, 18.1089}}	{110.351, 336.488}	inf	50.	143.458	{24., 302.}	212.361	0.456087	newly	113.	104.153	{15., 197.}	67.5147	0.409334	Reff	1.20861	2.54888	{2.10023, 2.93049}	110.894	ar	3086.	8963.47	{8362., 9399.}	190.456
26	R0	{1.18747, 5.88083}	{1.45235, 5.67739}	{{1.44485, 5.65591}, {1.454, 5.65591}}	{22.3066, 3.84233}	inf	178.	317.486	{180., 498.}	83.222	0.248021	newly	234.	352.135	{183., 559.}	62.1044	0.203155	Reff	1.02301	0.811047	{0.684834, 1.04106}	23.1309	ar	3614.	5459.16	{5218., 5711.}	51.056
27	R0	{1.29023, 4.76202}	{1.77933, 10.9709}	{{1.75469, 10.2835}, {1.76659, 10.896}}	{37.9081, 130.384}	inf	206.	591.3	{109., 1068.}	201.828	0.47383	newly	330.	416.733	{90., 706.}	60.1313	0.285281	Reff	1.17217	1.48517	{1.12441, 1.7332}	28.5822	ar	4245.	7261.88	{7019., 7451.}	71.0691
28	R0	{1.2258, 12.9056}	{1.51065, 15.8377}	{{1.51444, 16.0011}, {1.51444, 16.0011}}	{23.2381, 22.7196}	inf	188.	438.484	{256., 637.}	135.81	0.34993	newly	113.	189.645	{112., 279.}	72.0525	0.2158	Reff	1.0369	0.856409	{0.708758, 1.06056}	22.8526	ar	3639.	5920.45	{5692., 6120.}	62.6945
29	R0	{1.34702, 6.29049}	{1.81626, 9.6414}	{{1.70777, 8.49961}, {1.95639, 11.2959}}	{34.8351, 53.2694}	inf	264.	828.98	{304., 1308.}	219.4	0.457733	newly	312.	644.882	{280., 925.}	113.43	0.310665	Reff	1.19265	1.3692	{0.995648, 1.68874}	21.9388	ar	4560.	7341.06	{6791., 8035.}	60.9881
30	R0	{1.37931, 6.78741}	{1.66972, 7.81032}	{{1.47651, 5.4396}, {1.77826, 9.06807}}	{21.0545, 27.7795}	inf	251.	748.367	{296., 1141.}	198.821	0.43202	newly	287.	627.245	{317., 861.}	120.131	0.322354	Reff	1.17614	1.00823	{0.711457, 1.45997}	24.8968	ar	4667.	6735.86	{5606., 7304.}	44.3295
31	R0	{1.16062, 4.47529}	{1.43188, 3.82283}	{{1.36035, 3.22945}, {1.5451, 4.64686}}	{23.3717, 18.7764}	inf	80.	181.548	{47., 376.}	151.452	0.483447	newly	156.	415.032	{139., 766.}	180.521	0.542745	Reff	1.12661	1.32648	{1.20105, 1.48428}	19.0039	ar	3037.	4922.39	{4308., 5923.}	74.7799
32	R0	{1.37647, 18.7122}	{1.62413, 16.9784}	{{1.59192, 15.4649}, {1.63728, 17.5966}}	{17.9921, 9.26558}	inf	360.	597.488	{363., 834.}	70.2972	0.22068	newly	137.	238.86	{144., 345.}	77.6099	0.233748	Reff	1.09897	0.845827	{0.642976, 1.0662}	27.803	ar	4824.	6531.7	{6243., 6750.}	35.4
33	R0	{1.35084, 6.08841}	{2.28386, 16.6237}	{{2.01902, 13.4708}, {2.50427, 19.3027}}	{69.0694, 173.039}	inf	217.	821.986	{270., 1464.}	287.064	0.529342	newly	297.	402.169	{135., 711.}	66.2636	0.25946	Reff	1.23859	1.91899	{1.50597, 2.32181}	54.9341	ar	4759.	8532.32	{7916., 8942.}	79.2882
34	R0	{1.36912, 6.316}	{1.43783, 5.45881}	{{1.42659, 5.32185}, {1.454, 5.65591}}	{5.01841, 13.5716}	inf	198.	464.935	{312., 583.}	136.413	0.358584	newly	262.	579.774	{362., 721.}	121.509	0.331192	Reff	1.2459	1.01496	{0.873929, 1.25483}	19.4469	ar	4537.	5393.23	{5134., 5606.}	18.8721
35	R0	{1.35831, 7.09038}	{1.87467, 10.0414}	{{1.85376, 9.75343}, {1.88056, 10.2502}}	{38.015, 41.6194}	inf	331.	1130.02	{778., 1368.}	242.271	0.521909	newly	339.	809.592	{639., 963.}	139.829	0.373307	Reff	1.17304	1.16042	{0.816361, 1.54356}	18.4245	ar	4667.	7586.65	{7391., 7751.}	62.5595
36	R0	{1.28315, 5.89938}	{1.47115, 4.72074}	{{1.40116, 4.24222}, {1.54465, 5.30296}}	{14.6514, 19.9791}	inf	255.	466.912	{311., 667.}	87.5548	0.265205	newly	336.	704.265	{509., 935.}	111.668	0.314541	Reff	1.15291	1.03936	{0.828646, 1.26445}	14.6212	ar	4460.	5546.85	{4986., 6300.}	24.3689
37	R0	{1.19303, 2.75113}	{1.39414, 3.45421}	{{1.36035, 3.22945}, {1.50263, 4.22172}}	{16.8572, 25.5559}	inf	55.	323.571	{100., 505.}	494.805	0.738377	newly	184.	689.536	{214., 949.}	281.658	0.587012	Reff	1.13565	1.18365	{0.957822, 1.45214}	11.1015	ar	3142.	5264.25	{4699., 6134.}	67.5446
38	R0	{1.36033, 7.8088}	{1.47651, 5.4396}	{{1.47651, 5.4396}, {1.47651, 5.4396}}	{8.54057, 30.3401}	inf	249.	472.407	{329., 589.}	97.3077	0.302999	newly	256.	605.148	{439., 755.}	139.916	0.372926	Reff	1.22334	1.0556	{0.821383, 1.29844}	16.4203	ar	4941.	5655.22	{5391., 5906.}	14.455
39	R0	{1.27353, 3.70845}	{1.31707, 3.48629}	{{1.31535, 3.47086}, {1.31535, 3.47086}}	{3.41855, 6.56645}	inf	114.	263.647	{82., 378.}	144.995	0.407372	newly	257.	489.235	{190., 747.}	103.41	0.325145	Reff	1.17827	1.00111	{0.825777, 1.25484}	17.1071	ar	3725.	4536.88	{4051., 4928.}	21.7955
40	R0	{1.19983, 12.1486}	{1.54721, 18.4096}	{{1.54363, 18.3794}, {1.54363, 18.3794}}	{28.9522, 51.5372}	inf	191.	577.567	{289., 712.}	205.358	0.474659	newly	121.	217.4	{125., 272.}	85.1791	0.270048	Reff	1.02525	0.972611	{0.730754, 1.28492}	18.5821	ar	3619.	6130.	{5825., 6395.}	69.3838
41	R0	{1.20935, 2.67169}	{1.26964, 1.91763}	{{1.27121, 1.91565}, {1.27121, 1.91565}}	{5.04048, 28.2241}	inf	53.	188.136	{121., 256.}	258.919	0.538223	newly	165.	663.727	{351., 917.}	306.667	0.588855	Reff	1.16702	1.02175	{0.907008, 1.1503}	12.509	ar	3429.	3803.	{3404., 4233.}	13.2453
42	R0	{1.23313, 4.74781}	{1.3726, 5.11339}	{{1.3618, 4.98002}, {1.44485, 6.00596}}	{11.31, 7.70002}	inf	138.	297.136	{194., 449.}	115.316	0.311164	newly	211.	378.909	{257., 502.}	79.5778	0.238168	Reff	1.10217	0.950058	{0.777724, 1.21105}	18.4639	ar	3835.	4890.73	{4499., 5487.}	27.5287
43	R0	{1.26105, 2.39713}	{1.31792, 3.494}	{{1.31535, 3.47086}, {1.31535, 3.47086}}	{4.51007, 45.7577}	inf	88.	237.882	{32., 404.}	192.513	0.50963	newly	321.	513.882	{80., 797.}	85.7431	0.349124	Reff	1.20809	1.14175	{0.987433, 1.28799}	8.27691	ar	3723.	4536.88	{4051., 4928.}	21.8609
44	R0	{1.36948, 8.88006}	{1.75013, 9.51872}	{{1.58886, 7.29751}, {1.81647, 10.5184}}	{27.795, 13.9206}	inf	274.	610.51	{160., 983.}	144.368	0.387422	newly	232.	488.569	{136., 805.}	130.874	0.366057	Reff	1.23623	1.44157	{1.09836, 1.74181}	22.4406	ar	4437.	7179.08	{6567., 7492.}	61.8003
45	R0	{1.34882, 3.3791}	{1.93733, 6.87786}	{{1.47488, 3.83841}, {2.79531, 12.4651}}	{43.6318, 103.658}	inf	49.	185.404	{22., 377.}	296.978	0.506403	newly	143.	283.731	{36., 690.}	131.791	0.373646	Reff	1.32279	1.94965	{1.41512, 2.79682}	48.4395	ar	4422.	7516.88	{5512., 9252.}	69.9883
46	R0	{1.23774, 4.34917}	{1.31023, 3.04996}	{{1.31023, 3.04996}, {1.31023, 3.04996}}	{5.85664, 29.8726}	inf	107.	234.529	{159., 324.}	123.914	0.34212	newly	199.	559.059	{406., 754.}	180.934	0.431041	Reff	1.16533	1.06237	{0.889646, 1.20043}	10.3518	ar	3685.	4271.65	{3848., 4506.}	15.9199
47	R0	{1.34327, 4.78811}	{1.50431, 5.66417}	{{1.38889, 4.25615}, {1.68297, 7.5059}}	{11.9883, 24.7404}	inf	102.	205.081	{25., 525.}	139.322	0.541502	newly	151.	291.973	{39., 582.}	132.629	0.543109	Reff	1.29693	1.48388	{1.22006, 1.84315}	18.0806	ar	4517.	5523.11	{4580., 7688.}	38.3225
48	R0	{1.24366, 4.25814}	{1.98594, 12.5416}	{{1.80156, 10.0119}, {2.16755, 14.8261}}	{59.6853, 194.532}	inf	131.	513.333	{165., 1020.}	291.965	0.51063	newly	260.	345.982	{134., 741.}	60.9109	0.216294	Reff	1.16755	1.76579	{1.4474, 2.02796}	51.6449	ar	3678.	7901.56	{7268., 8433.}	114.833
49	R0	{1.36017, 7.5415}	{1.32029, 4.26982}	{{1.31748, 4.25564}, {1.31748, 4.25564}}	{2.93221, 43.3823}	inf	127.	177.318	{104., 259.}	53.0064	0.180486	newly	116.	266.045	{151., 423.}	133.346	0.333191	Reff	1.22946	0.872924	{0.768486, 1.10998}	28.9992	ar	4178.	4380.86	{3968., 4795.}	6.52661
50	R0	{1.25333, 6.63578}	{1.41571, 4.94504}	{{1.41571, 4.94504}, {1.41571, 4.94504}}	{12.9559, 25.4791}	inf	201.	202.714	{107., 329.}	38.8534	0.197595	newly	235.	245.	{116., 456.}	44.2958	0.214047	Reff	1.0642	0.777407	{0.707855, 0.916106}	27.1862	ar	3401.	5143.62	{4811., 5368.}	51.2384
